# Open data in a deeply connected world

**DOI:** 10.1186/s13059-020-02010-6

**Published:** 2020-04-20

**Authors:** Barbara Cheifet

**Affiliations:** BioMed Central, New York, NY USA

We are currently living and working in an era where huge amounts of data are generated every day. Questions obviously arise over where to put this data, who owns it, and who can use it. It is clear that giving open access to research can be beneficial for the community. For example, ever since the emergence of COVID-19, a contagious respiratory disease that is induced by infection of SARS-CoV-2, there have been over 1515 peer-reviewed research articles and commentaries published in 2 months. Publishers are working day and night to expedite the publication of such knowledge and to make the articles freely open to the public. In addition to free access, Springer Nature has collated all research on this topic into an easily accessible resource online: https://www.springernature.com/gp/researchers/campaigns/coronavirus.

It is clear that the rapid advancement of knowledge on this specific disease and specific coronavirus was fueled by the open attitude and willingness of researchers to share raw sequencing data of the virus and their research findings, and this has encouraged other researchers to share and build on the data. According to the GISAID website (https://www.gisaid.org/epiflu-applications/next-hcov-19-app/), there are 1249 SARS-CoV-2 sequences available and the numbers are growing rapidly along with the emerging pandemic. Researchers are also sharing their research on preprint platforms such as medRxiv and bioRxiv to more quickly disseminate their results to the public. The speed at which this new open data has disseminated worldwide shows how clearly important to the community open access and open data is. It is also evident to us that, being a proponent of making raw research data open and available, *Genome Biology* needs to reiterate that all datasets on which the conclusions of the paper rely should be available to readers, and where there is a community established norm for data sharing.

## Clarifying our open data policy

*Genome Biology* has very strict open access, open source, and open data policies. As we all know, scientific research is a process of trial and error, and so is scientific publication. Even though journals rely on peer experts in the field to ensure the robustness of the analyses and minimize oversights, the evolving nature of scientific research means that given time, many studies could be contradicted or proven to be flawed. This is of course not because authors intend to mislead, but it is possible that a minor raw data pre-processing step or pre-assumption could potentially affect the final conclusions. Therefore, it is important to have the raw data available and similarly the source code and the implementation of the analyses. A stable and reproducible tool is the foundation of reproducible results.

To encourage data availability to all researchers, *Genome Biology* accepts a broad list of community-accepted repositories, which are found here: https://www.nature.com/sdata/policies/repositories#general. These repositories are all indexed in the International Nucleotide Sequence Database Collaboration (INSDC), which is a long-standing initiative established in 2002 between the United State’s Genbank (NCBI), the DNA Database of Japan (DDBJ), and the European Nucleotide Archive (EMBL-EBI) and is an example of a worldwide scientific collaboration. They have been proven to be stable and resilient over time, through funding shifts and other potentially disruptive situations, and the data within them is always available to the public. These databases are regulated by an international advisory board and updated synchronously.

Of note, a few China-based genome data repositories, such as the National Genomics Data Center (https://bigd.big.ac.cn/), are not currently indexed with INSDC, and therefore, *Genome Biology* does not accept them as the sole repository for data in our publications. If the data deposited in one of these repositories is open access, by definition, it should also be able to be deposited in another, INSDC-indexed, repository. We are aware of discussions with these repositories and the INSDC.

This does not mean that we do not share concerns regarding patient privacy in clinical genomics data. Aside from pushing the privacy protection technologies in genomic data sharing [1, 2], *Genome Biology* accepts gated repositories for human genomics data. We acknowledge that different regions and funding bodies may have their own specific requests for how human data should be handled and made available, and we do honor these as much as possible. Authors have an ethical and legal responsibility to respect participants’ rights to privacy and their identities.

We understand regional data regulation should be interpreted in a case by case manner, as data from different populations commonly have different codes for repository requirement and safety concerns. We urge our authors to read and understand their regional data regulations specific to their own project before preparing manuscripts; this includes a few European regions and China [3, 4]. For instance, according to the latest version of *Regulations of the People’s Republic of China on the management of human genetic resources* [4], China continues to encourage institutions, institutes, medical centers, and companies to engage in international collaboration of researches involving human genetic resources. However, to make sure the pertinent data can be shared in a regulation-compliant manner, authors are required to file the data sharing plan and have it recorded online by the relevant authorities first.

## Growing influence of preprints

It is also clear to us that preprint servers are becoming more popular in the sciences, and for good reason. There are many benefits to posting a research or method article on a preprint, including faster dissemination of results or useful methods to the community, and opportunities for feedback prior to peer review. *Genome Biology* fully supports and encourages preprint posting of papers for the main reason that it encourages openness of research and more scientific discussion. We encourage our authors to post to a preprint server such as bioRxiv (http://www.biorxiv.com) when we send a paper for review; however, we notice an increasing number of papers that are submitted directly from bioRxiv using B2J, or already posted on a preprint at the time of submission. As mentioned in a recent publication [5], *Genome Biology* is among the journals that publish a large number of papers that were previously posted on bioRxiv, and we would be happy to see this trend continue.

## Recognizing the downsides

With all of the benefits of preprint sharing and open data, there are still some limitations that should also be addressed. For example, some gated repositories can require time to accept and make data available, and getting access to such data may be difficult. Concerns for patient privacy also require consideration. With the recent dissemination of research related to COVID-19, we have seen that preprint results have been misinterpreted by the media [6]. On the contrary to its original goal of saving lives and assisting public health response, these waves of misinterpretation inspired public panic and anger against scientists who are working hard for public safety.

We feel that it is the role of journals to provide sound peer review to safeguard data reproducibility and reliability. In order to ensure this, we need peer reviewers to be able to access raw data and source code, which is why we ask for data to be deposited at early stages of submission. In cases where the results in a paper are contradicted after publication, having source code and data available can make issues easier to resolve.

## Conclusions

In this era of “big data,” we need to be able to disseminate data quickly to others who can build upon it and reproduce it. This means that we have to make sure that data is freely available in a secure repository for readers and reviewers. Of course, for at least the foreseeable future, clinical, identifiable data needs to be deposited with caution, but we expect that progress will be made in this area in the years to come. We also realize that our current requirement to accept only INSDC-indexed repositories can result in inconvenience to some. We hope that we will be able to accept additional repositories in the near future. We highly recommend that you consider what repository to place your data in before or at a very early point in your submission. If you are unsure of which repository to use, our editors are always available for discussion.
